# Research in practice: Immune checkpoint inhibitor related autoimmune bullous dermatosis

**DOI:** 10.1111/ddg.15638

**Published:** 2025-02-13

**Authors:** Jasper N. Pruessmann, Wiebke Pruessmann, Christian D. Sadik

**Affiliations:** ^1^ Department of Dermatology University of Luebeck University Hospital Schleswig‐Holstein Luebeck Germany

**Keywords:** autoimmune dermatosis, Immune checkpoints, immune related adverse event, pemphigoid

## Abstract

Immune checkpoint receptors and ligands such as cytotoxic T lymphocyte antigen‐4 (CTLA‐4), programmed death‐1 (PD‐1) and ligand‐1 (PD‐L1) are widely expressed on immune and non‐immune cells and fine tune the activation level of immune cells, thus, enabling, preventing, or terminating immune responses. Blockade of CTLA‐4, PD‐1 or PD‐L1 by checkpoint inhibitors (CIs), unleashing immune responses, has become a mainstay in the treatment of diverse types of cancer. The induction of autoinflammatory, yet unspecific tissue damage in diverse organs is called an immune related adverse event (irAE), a class side‐effect of CIs and may require the discontinuation of immunotherapy. Among frequent skin rashes, CIs targeting the PD‐L1/PD‐1 axis can elicit the IgG autoantibody‐ and granulocyte‐driven bullous pemphigoid (BP) in about 0.3% to 0.6% of treated patients. Pathogenesis of BP requires a complex cellular inflammatory response after anti‐BP180 autoantibody binding to the dermal epidermal junction. The prevalence of autoantibodies against BP180 in healthy blood donors of approximately 0.52% equals the prevalence of irBP among treated cancer patients, underlining the potential relevance of the PD‐1 mediated regulation of tissue inflammation for spontaneous BP. If skin rashes appear during CI therapy, biopsies should be taken and examined by histopathological and direct immunofluorescence microscopy.

## CLINICAL RELEVANCE

Immune checkpoints—co‐stimulatory and inhibitory receptors along with their ligands—are broadly expressed on both immune and non‐immune cells, serving as crucial regulators of immune responses. More specifically, immune checkpoints fine‐tune the activation levels of immune cells, enabling, preventing, or terminating immune responses. They play a pivotal role in determining whether to initiate an immune response or maintain tolerance and tissue homeostasis.

The immune checkpoints programmed death 1 (PD‐1) and its ligand 1 (PD‐L1) and cytotoxic T lymphocyte antigen‐4 (CTLA‐4) are the most prominent checkpoint inhibitory molecules (CIMs) and directly control the activation level of T cells.

CTLA‐4 is a co‐inhibitory competitor of the T cell co‐stimulatory receptor CD28, which provides the second signal to T cell receptor signaling. CTLA‐4 restricts the stimulation of naive T cells by binding and blocking CD80 and CD86, the ligands of CD28, which are expressed by antigen presenting cells (APC).^1^ CTLA‐4 is expressed after activation of naïve T cells and constitutively on regulatory T cells. A defect in CTLA‐4 leads to lymphoproliferative disease accompanied by autoantibody mediated cytopenias, organ‐specific autoimmunity, lymphadenopathy, and splenomegaly, lymphocytic infiltration of nonlymphoid organs and B cell exhaustion caused hypogammaglobulinemia with increased susceptibility to infection.^2^ PD‐1 is induced by continuous TCR signaling. Its ligand PD‐L1 is expressed by various immune and non‐immune cell types in steady state and after induction.^1^ Therefore, PD‐1 serves as an immune checkpoint for T cells during almost all cell contacts. To date, a defect of PD‐1 has been described in only a single human being. This patient suffered from autoimmune reactions in different organs and a defect in the protection from tuberculosis. A severely disrupted lymphocyte homeostasis has been observed, characterized by the depletion and dysfunction of specific T‐cell and natural killer cell subsets, alongside the proliferation of nonspecifically activated, cytokine‐releasing T cells.^3^ Taken together, immune checkpoints provide stimulatory or inhibitory co‐signaling to T‐cell receptor activation, establishing co‐inhibitory receptors and their ligands as central regulators of T‐cell‐driven immune responses.

Blockade of CTLA‐4, PD‐1 or PD‐L1 by checkpoint inhibitors (CIs), unleashing immune responses, has become a mainstay in the treatment of diverse types of cancer. The induction of autoinflammatory, yet unspecific tissue damage in diverse organs which mostly affect the skin, gut, lung or endocrine glands are called immune related adverse events (irAE), a class side‐effect of CIs.^4^, ^5^ Although the emergence of irAEs is associated with a better response to CI treatment, it may unfortunately also require the discontinuation of CIs.^6^ CIs targeting the PD‐L1/PD‐1 axis have been described to elicit a specific IgG autoantibody‐ and granulocyte‐driven inflammatory disease of the skin, namely bullous pemphigoid (BP), in about 0.3% to 0.6% of treated patients.^7^, ^8^, ^9^, ^10^, ^11^

In BP, autoantibodies bind structural proteins of the adhesion complex at the dermal‐epidermal junction (DEJ), more specifically BP180 (type XVII collagen), and/or BP230 (dystonin, part of hemidesmosomes). This initiates the effector phase of the disease with an inflammatory cascade of complement activation, granulocyte attraction and release of proteases and other lytic compounds leading to the destruction of the DEJ and blister formation.^12^ Patients are mostly above 75 years of age at the time of disease manifestation without a strong sex preference and the overall prevalence of BP arises to 200/million/year in individuals over 80 years of age.^13^ Factors frequently associated with disease manifestation appear to be neurological and psychiatric disorders (e.g., Parkinson's disease, dementia, uni‐/bipolar disorder)^14^ and drugs, such as diuretics, antipsychotics, and dipeptidyl‐peptidase IV inhibitors (gliptins).^12^, ^13^ The typical skin lesion usually shows a progression from erythema, urticarial plaque, blister, erosion to hyper‐ or hypopigmented re‐epithelized macula without scarring and appear in different regions of the body at different stages at the same time. However, BP is a clinically heterogenous disease as all stages of skin eruptions may be observed exclusively while severe itch is a characteristic symptom in most BP patients; itch, on the other hand, may also be the only symptom in so‐called premonitory BP.^13^ Premonitory, classical bullous and non‐bullous BP have several clinical differential diagnoses, such as scabies, bullous drug reactions or eczema, that require specific therapies. The gold standard for diagnosis of BP is a perilesional biopsy and direct immunofluorescence staining of tissue bound autoantibodies, which cause a linear staining at the basement membrane. A lesional biopsy for histopathological examination is required to exclude differential diagnoses. Furthermore, detection of circulating autoantibodies in the blood of patients is required to identify the target antigen, which helps to discriminate different pemphigoid diseases, and may be used to monitor disease activity. Therapy of BP is based on immunosuppressants and includes corticosteroids systemically and/or (whole‐body) topical administration of superpotent corticosteroids.^15^ Mortality was reduced from 70% during the first flare of BP in pre‐immunosuppressant era to 2–3 fold increased overall mortality over age and sex matched controls by adequate treatment.^13^ Mortality is often associated with infections.

The specificity and frequency of checkpoint therapy induced BP as a side effect suggest that PD‐L1/PD‐1 are of importance in the pathophysiology of BP. However, the roles of PD‐L1/PD‐1 in autoantibody‐ and granulocyte‐driven immune responses, as well as the involvement of T cells in bullous pemphigoid (BP), remain poorly understood. This is despite the widespread expression of PD‐L1 and PD‐1, including on granulocytes, and their presence in the inflamed skin of BP patients.^16^

## HYPOTHESIS

**FIGURE 1:**
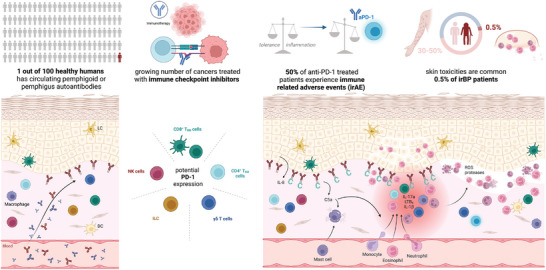
Proposed pathophysiology of immunotherapy related bullous pemphigoid. Roughly 1% of healthy humans are asymptomatic carriers of circulating, potentially skin‐bound, pemphigoid or pemphigus related non‐pathogenic autoantibodies. In healthy skin, low numbers of resident immune cells are present. We hypothesize that CD8^+^ TRM cells, CD4^+^ TRM cells, γδ T cells, innate lymphoid cells (ILC), and natural killer (NK) cells might express PD‐1 on their cell surface in steady state conditions. Upon pharmacological PD‐1 inhibition, these cells might be activated, release cytokines and facilitate Fc receptor‐independent events leading to the release of interleukin 8 (IL‐8) from basal keratinocytes. Complement is activated and bound at the dermal‐epidermal junction (DEJ) which leads to mast cell degranulation. Chemokine gradients (IL‐17a, LTB4, IL‐1β) promote the infiltration of effector cells such as monocytes, neutrophils and eosinophils into the upper dermis. Granulocytes at the DEJ release proteases and reactive oxygen species (ROS) leading to subepidermal blister formation in 0.3% and 0.6% of patients treated with anti‐PD‐1 checkpoint inhibitors developing irBP (Created with BioRender.com).

The occasional manifestation of BP under PD‐1/PD‐L1 inhibition suggests that the PD‐1/PD‐L1 axis may counteract the emergence and severity of spontaneously emerging BP. It is conceivable that this occurs at the level of tolerance breakdown against BP180, leading to the generation of pathogenic antibodies, as well as at the level of the induction and counter‐regulation of tissue inflammation. In healthy blood donors (age range 18–69 years), the combined prevalence of the most frequent skin‐specific autoantibodies (anti‐desmoglein 1, ‐desmoglein 3, ‐BP180, ‐BP230) is roughly 1% (Figure 1).^17^ While desmoglein specific antibodies lead to acantholysis by simply binding their antigens, pathogenesis of pemphigoid diseases requires a complex cellular inflammatory response after antibody binding. It is important to note that pemphigus diseases have rarely been reported as immune‐related adverse events (irAEs) to date,^18^ suggesting that the development of pathogenic autoantibodies is less likely to be triggered by CTLA‐4 or PD‐1/PD‐L1 immune therapy. Second, the prevalence of autoantibodies against BP180 in healthy blood donors is approximately 0.52%, though potentially higher in older individuals, but still close to the prevalence of irBP among treated cancer patients,^17^, ^19^ highlighting the potential relevance of the PD‐1 mediated regulation of tissue inflammation for spontaneous BP.

The IgG production by B cells in B cell follicles of lymph nodes is dependent on the help of specific T cells called T follicular helper (Tfh) cells. This indicates that the production of autoantibodies in the pre‐clinical phase of a disease already requires activation of autoreactive T cells. Compared to PD‐1/PD‐L1 inhibition, BP associated with anti‐CTLA‐4 monotherapy has been described in few case reports only although irAE of CTLA‐4 inhibition are more frequent and often more severe compared to those of PD‐1/PD‐L1 inhibition.^6^, ^20^ While CTLA‐4 inhibition is likely to enhance the activation of naïve, self‐reactive T cells by APCs, interruption of the PD‐1/PD‐L1 axis might primarily disturb peripheral tolerance by activating effector and memory T cell subsets.^1^ The role of T cells and PD‐1 in the pathogenesis of BP has yet to be fully understood but Tfh and skin resident memory T cells express PD‐1 and are potentially involved.^21^ As almost half of the patients develop eczematous or lichenoid skin rashes under checkpoint therapy (Figure 1), we hypothesize that pre‐existing skin‐bound anti‐BP180 autoantibodies direct the inflammatory response, induced by activated PD‐1 expressing (autoreactive) T cells in the skin, into a granulocyte dominated inflammation and destruction of the dermal‐epidermal junction. To test this hypothesis, we are studying the role of PD‐1 inhibition specifically in the effector phase of the autoantibody induced and granulocyte driven immune response of pemphigoid disease.

## RESEARCH FOR CLINICAL PRACTICE

We have generated preliminary data in a mouse model of the effector phase of pemphigoid diseases indicating PD‐1 and PD‐L1 counteract skin inflammation in this model. Thus, skin inflammation was aggravated when PD‐1 was genetically ablated or pharmacologically inhibited. These findings support the notion that CI treatment may promote the emergence of BP at the level of the effector phase. Furthermore, activation of PD‐1/PD‐L1 may be a promising therapeutic approach to treat spontaneous BP. We are currently investigating this treatment concept as part of a research project funded by the DDG (German Dermatological Society) and the ADF (Consortium for Dermatological Research).

The importance of PD‐1 signaling to maintain peripheral tolerance by limiting autoreactive T cell activity in the skin has also been associated with lichenoid irAE. Damo et al. developed an antigen model system with mouse epidermis expressing a T cell specific model‐antigen. They showed that even though CD8 T cells specific for this antigen acquire an effector phenotype, no pathology is observed. In fact, the cells accumulate in the dermis in steady state but do not reach their epidermal target cells because PD‐1 signaling prevents these cells from migrating into the epidermis, releasing cytokines, and thereby causing local pathology. Patients suffering from PD‐1 therapy induced lichenoid skin eruption indeed carry clonally expanded effector CD8 T cells in lesional and non‐lesional skin.^22^

## CONCLUSIONS FOR CLINICAL PRACTICE

Understanding the role of immune checkpoints and targeting them has represented a significant breakthrough in the treatment of malignant diseases. The new class of immune related adverse events affects almost half of the treated patients. Defects of immune checkpoints in human patients are represented by case studies only, which nevertheless helped to improve understanding their function. Even though inhibitory checkpoint therapy is in clinical use for about a decade, the CTLA‐4 agonist Abatacept still is the only CIM activator on the market to treat autoimmune diseases. Though, it does pose the risk of severe, potentially fatal infections. However, the development of PD‐1 agonists for the treatment of autoimmune diseases has not been successful yet, which might be due to the abundance of PD‐L1 presentation providing peripheral tolerance, likely even in autoimmune disease.^23^ The multifaceted ways of how patients respond to checkpoint therapy with skin related irAE indicate that the individual context greatly influences the direction of inflammation reaching from frequent pruritic, lichenoid, eczematous, psoriasiform to rare humoral and granulocytic dominated bullous immune responses.^24^ Identifying key cytokines driving these different responses is necessary to enable targeted treatment of irAE, without losing benefit in cancer immunotherapy, and likewise treatment of autoimmune diseases alternatively to immune checkpoint agonists and especially general immunosuppressants. If skin rashes appear during CI therapy, biopsies should be considered and taken from lesional and perilesional skin to be examined by histopathological and direct immunofluorescence microscopy, respectively.

## FUNDING

Return fellowship PR 1652/2‐1 (German research foundation [DFG]), Juniorförderung J03‐2020 (University of Luebeck), clinical research unit 303 (DFG), collaborative research center 1526 (DFG), Miniproposal 2021 (Cluster of exellence 2167, DFG). J.P. is furthermore supported by the Clinician Scientist Program of the Deutsche Stiftung Dermatologie e.V. (Deutsche Dermatologische Gesellschaft e.V. [DDG; https://derma.de/stipendien‐forschungspreise]/Arbeitsgemeinschaft Dermatologische Forschung e.V. [ADF; https://www.adf‐online.de]). The Clinican Scientist Program of the DDG is kindly supported by: Abbvie Germany, Allmirall, Janssen‐Cilag, LEO Pharma, Lilly, Sanofi and UCB Pharma.

## CONFLICT OF INTEREST STATEMENT

None.
